# Transfusion burden in non-dialysis chronic kidney disease patients with persistent anemia treated in routine clinical practice: a retrospective observational study

**DOI:** 10.1186/1471-2369-13-5

**Published:** 2012-01-24

**Authors:** Kathleen M Fox, Jerry Yee, Ze Cong, John M Brooks, Jeffrey Petersen, Lois Lamerato, Shravanthi R Gandra

**Affiliations:** 1 Strategic Healthcare Solutions, LLC, P.O. Box 543, Monkton, MD 21111, USA; 2 Division of Nephrology and Hypertension, Henry Ford Health System, 2799 W. Grand Blvd, Detroit, MI 48202, USA; 3 Global Health Economics, Amgen, Inc., 1 Amgen Center Drive, Thousand Oaks, CA 91320, USA; 4 College of Pharmacy, University of Iowa, 115 S. Grand Avenue, Iowa City, IA 52242, USA; 5 Clinical Research, Amgen, Inc., 1 Amgen Center Drive, Thousand Oaks, CA 91320, USA; 6 Josephine Ford Cancer Center, Henry Ford Health System, 1 Ford Place Center, Detroit, MI 48202, USA

## Abstract

**Background:**

Transfusion patterns are not well characterized in non-dialysis (ND) chronic kidney disease (CKD) patients. This study describes the proportion of patients transfused, units of blood transfused and trigger-hemoglobin (Hb) levels for transfusions in severe anemic, ND-CKD patients in routine practice.

**Methods:**

A retrospective cohort study of electronic medical record data from the Henry Ford Health System identified 374 adult, ND-CKD patients with severe anemia (Hb < 10 g/dL and subsequent use of erythropoiesis-stimulating agents [ESA] therapy, blood transfusions, or a second Hb < 10 g/dL) between January 2004 and June 2008. Exclusions included those with prior diagnoses of cancer, renal or liver transplant, end-stage renal disease, acute bleeding, trauma, sickle cell disease, or aplastic anemia. A gap of ≥ 1 days between units of blood transfused was counted as a separate transfusion.

**Results:**

At least 1 transfusion (mean of 2 units; range, 1-4) was administered to 20% (75/374) of ND-CKD patients with mean (± SD) follow-up of 459 (± 427) days. The mean (± SD) Hb level closest and prior to a transfusion was 8.8 (± 1.5) g/dL. Patients who were hospitalized in the 6 months prior to their first anemia diagnosis were 6.3 times more likely to receive a blood transfusion than patients who were not hospitalized (p < 0.0001). Patients with peripheral vascular disease (PVD) were twice as likely to have a transfusion as patients without PVD (p = 0.04).

**Conclusions:**

Transfusions were prevalent and the trigger hemoglobin concentration was approximately 9 g/dL among ND-CKD patients with anemia. To reduce the transfusion burden, clinicians should consider other anemia treatments including ESA therapy.

## Background

Chronic kidney disease (CKD) affects about 26 million adults (11%) in the United States, with the early stages (stages 3-4) being approximately 100 times more prevalent than kidney failure [1]. Persistent anemia is a consequence of the declining endogenous erythropoietin production seen in progressive CKD [2]. Anemia of CKD can be successfully treated by administration of an erythropoiesis-stimulating agent (ESA) [3]. Blood transfusion in non-dialysis (ND) CKD occurs in nearly 10% of Medicare-insured CKD patients per year despite the availability of ESA therapy, a rate four times as great as in older patients without CKD [4]. The use of transfusion can correct anemia in the short term, but may also increase the risk of sensitization to HLA antibodies and thus delay or even preclude kidney transplant [5]. Additional risks associated with transfusion include iron overload, transfusion reactions, transmission of infectious agents, and acute lung injury [5].

Studies in CKD patients on dialysis have shown a declining pattern of transfusion use subsequent to the introduction and adoption of anemia management with ESAs [6]. However, in the ND-CKD setting, the pattern of use of transfusion among patients with CKD-related anemia has not been well characterized. The present study describes the proportion of patients transfused among severe anemic ND-CKD patients who were managed in routine clinical practice. The study objectives were to estimate the proportion of ND-CKD patients receiving transfusions, to estimate the exposure-adjusted transfusion rate, and to characterize the patients who received a transfusion, among severely anemic, ND-CKD patients.

## Methods

A retrospective observational cohort study was conducted using electronic medical record data collected by the Henry Ford Health System (HFHS). The Henry Ford Health System is a vertically integrated health care system providing clinical services to a diverse Michigan community, with over 2.5 million patient visits and 65,000 hospital admissions annually. HFHS is the largest integrated health system in southeast Michigan area and the patient population is relatively stable. Administrative data was linked to clinical, pharmacy, and laboratory data for eligible patients.

### Study population

All HFHS patients, 18 years of age or older, who had a diagnosis of CKD stage 3-5, and were not on dialysis during the study intake period of January 1, 2004 and June 30, 2008 were identified. A diagnosis of CKD stage 3-5 was defined as the presence of at least 2 ICD-9 codes for CKD (585.xx, 403.xx, 404.xx) within 6 months and an eGFR ≥ 15 and < 60 mL/min/1.73 m^2 ^estimated using the CKD-EPI equation [1]. Two or more ICD-9 codes for CKD were required to rule out patients who might have received 1 ICD-9 code during the initial work-up of the diagnosis.

To be included in the study, these CKD patients had to have severe anemia that was defined, for the purpose of this study, as hemoglobin (Hb) < 10 g/dL and at least one of the following subsequent events (within 6 months): 1) received ESA therapy, 2) had a blood transfusion, or 3) second Hb < 10 g/dL. The criterion of at least one additional event (ESA therapy, transfusion, or second Hb < 10 g/dL) was used to include patients with non-acute, severe anemia that was related to CKD. The index date was the date of the first Hb < 10 g/dL, i.e., first observed date with an indication of severe anemia. Patients with the following conditions 6 months prior to the index date or any time during follow-up were excluded from the analysis in an attempt to eliminate reasons for transfusion other than CKD-related anemia: cancer, acute bleeding episodes, gastrointestinal surgery, sickle cell or aplastic anemia, recent trauma and liver transplant. Patients who had end stage renal disease (ESRD), or received kidney transplant, hemodialysis or peritoneal dialysis prior to the index date were also excluded. The follow-up period began with the index date (date of first Hb < 10 g/dL) and not the subsequent event of ESA therapy, transfusion, or second Hb < 10 g/dL since this event may occur at any time during the 6 months after the index date. Eligible patients were followed until one of the following events transpired in the follow-up period: 1) initiation of dialysis or development of ESRD (diagnostic code), 2) renal transplantation, 3) death, or 4) last date of data capture, i.e. December 31, 2009.

### Study variables

The study outcome was any blood transfusion (inpatient or outpatient) occurring during the follow-up period. A transfusion was defined as all units of blood transfused on consecutive days; a gap of 1 or more days between transfused units of blood was counted as a separate transfusion. This definition was applied to account for patients who had a transfusion order late in the day such that the last unit of blood was transfused on the following day. Exposure-adjusted transfusion rate was calculated as the total number of transfusions divided by total person time during follow-up and reported per 100 patient-years. This rate was calculated to adjust for the varying length of follow-up among patients since some patients may have died, progressed to ESRD, started dialysis or dropped out.

The number of units given during each transfusion was summarized. Baseline hemoglobin was the hemoglobin concentration 30 days preceding the index date. The post-index Hb concentration closest to but prior to (within 2 days) the transfusion was considered the trigger Hb for the transfusion. Comorbid conditions (i.e. cardiovascular disease, peripheral vascular disease, diabetes, hypertension, dyslipidemia, cerebrovascular disease) were captured in the 6 months prior to the index date. ICD-9 diagnosis codes in the primary or secondary position on any non-diagnostic claim were used to identify comorbid conditions. Occurrence of transfusion, care visit to a nephrologist, and hospitalization in the 6 months prior to the index date were recorded.

The study was approved by the Henry Ford Health System Institutional Review Board.

### Statistical analysis

Means and variances were calculated for continuous variables and number and percent of patients for categorical variables. Univariate analysis compared the proportion of patients transfused for each patient characteristic (chi-square, *t *tests, and Mann-Whitney U test for median length of follow-up) and those characteristics that had a P < 0.10 were entered into a multivariable analysis. Multivariable logistic regression analysis was conducted to estimate the likelihood of a blood transfusion, adjusting for patient demographics and clinical characteristics. All variables were entered into the model simultaneously without a stepwise procedure. Variables included in the model were those that were demographic variables (age, gender, and race) and clinical characteristics assessing patient's health status at baseline (comorbid conditions and prior nephrologist visit) regardless of the significance level in the univariate analysis. Characteristics that were significantly associated with transfusion in univariate analysis (P < 0.10) were also included in the same model. Unique ICD-9 codes were used for the comorbid conditions of cardiovascular disease and peripheral vascular disease. Continuous covariates were stratified at the mean, below the mean vs. at or above the mean. Odds ratios and 95% confidence intervals were calculated.

## Results

**Figure 1:**
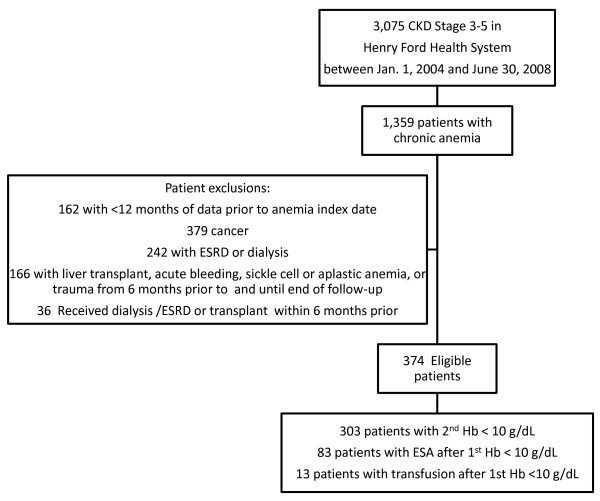
**Non-dialysis CKD patients with persistent anemia: inclusion and exclusion chart**.

There were 3,075 CKD patients stage 3-5 in the HFHS between January 1, 2004 and June 30, 2008. One-third of patients (32%) did not meet our definition for severe anemia, or had claims indicating the presence of ESRD or dialysis at the index date, a history of cancer, or other blood-related conditions that influence the likelihood of anemia (Figure 1). A cohort of 374 patients had non-dialysis, CKD with severe anemia and was included in the analysis. Among the 374 patients, 303 (81%) were included in the cohort because they had a second Hb < 10 g/dL which occurred, on average, within 15 days of the first Hb < 10 g/dL; 83 patients had an ESA prescribed within an average of 21 days after the first Hb < 10 g/dL, and 13 patients had a blood transfusion within an average of 5 days after the first Hb < 10 g/dL.

The cohort comprised mostly older patients (mean age of 71 years) and women (60%) with approximately 66% of the cohort having seen a nephrologist prior to the index date (Table 1). There were 75 patients who had at least 1 transfusion in the follow-up period.

**Table 1:** Characteristics of the Henry Ford cohort of non-dialysis CKD patients with persistent anemia

Characteristics	Total cohort N = 374
**Age, mean (SD)**	70.8 (12.9)

**Men, %**	39.6

**White, %**	45.7

**African American, %**	50.3

**eGFR at index date†, mean (SD)**	32.1 (12.7)

**Hemoglobin closest to index date (baseline), %**	

**< 10 g/dL**	8.5

**≥ 10 g/dL**	91.5

**Transfusion prior to index date, %**	3.5

**Cardiovascular disease, %**	36.6

**Diabetes, %**	59.1

**Peripheral vascular disease**	16.6

**Nephrologist visit prior to index date, %**	66.3

**Hospitalized prior to index date, %**	49.7

**Follow-up period, days, mean (SD)**	459.5 (427.4)

†index date is the date of the first Hb < 10 g/dL in the study identification period.

### Proportion transfused and transfusion rates

The proportion of patients who had a transfusion was 20% for the cohort; 14% of the cohort had only 1 transfusion, 3% had 2 transfusions and 3% had 3 or more transfusions. The proportion transfused was greater among patients with peripheral vascular disease (PVD) at baseline than among patients without PVD (29% vs. 18%, respectively, P = 0.05) (Table 2). In addition, more African American and other minority patients had a transfusion (25% and 27%, respectively) than white patients (14%) (P = 0.05).

**Table 2:** Proportion of patients with a transfusion by patient characteristics

Characteristic	Proportion of patients transfused, %	p-value
**Age**		0.95

**18-64 years, n = 105**	19	

**65-74 years, n = 99**	20	

**75 or older, n = 170**	21	

**Gender**		0.86

**Male, n = 148**	20	

**Female, n = 226**	20	

**Race**		0.054

**Caucasian, n = 171**	14	

**African American, n = 188**	25	

**Other races, n = 15**	27	

**Transfusion in the pre-index period**		0.09

**Yes, n = 13**	38	

**No, n = 361**	19	

**eGFR, mL/min/1.73 m^2^, in the pre-index period**		0.82

**< 15, n = 13**	15	

**15 to < 30, n = 135**	21	

**30 to < 45, n = 101**	19	

**45 to ≤ 60, n = 36**	22	

**Nephrologist visit in pre-index period**		0.31

**Yes, n = 248**	19	

**No, n = 126**	23	

**Hospitalization in pre-index period**		< 0.0001

**Yes, n = 186**	33	

**No, n = 188**	7	

**Comorbid condition in pre-index period**		

**Diabetes**		0.38

**Yes, n = 221**	19	

**No, n = 153**	22	

**Peripheral vascular disease**		0.053

**Yes, n = 62**	29	

**No, n = 312**	18	

**Cardiovascular disease**		0.14

**Yes, n = 137**	24	

**No, n = 237**	18	

A larger percentage of patients with a hospitalization during the 6-month pre-period had a transfusion compared to those without a prior hospitalization (33% vs. 7%, respectively, P < 0.0001, Table 2). Additionally, the frequency of patients who had a transfusion was larger among those with more hospital days before the anemia index date (more than 5 days over a 6-month period; mean number of hospital days was 5) compared to those with fewer hospital days (34% vs. 13%, respectively, P < 0.0001). Among patients who had a transfusion prior to their anemia index date, the proportion transfused during follow-up was larger than among patients who did not previously have a transfusion (38% vs. 19%, respectively, P = 0.09); however, only 13 patients had a prior transfusion. The percentage of patients who had a transfusion did not differ by CKD stage, gender, age, baseline eGFR, Hb prior and closest to index date, comorbid conditions of diabetes, hypertension, cardiovascular disease, dyslipidemia, and cerebrovascular disease, or nephrology visit prior to anemia index date.

The patient cohort had an average (± SD) length of follow-up of 459 (427) days. The average exposure-adjusted transfusion rate was 182.2 transfusions per 100 patient-years. The rate of transfusions was higher among patients who had a hospitalization prior to the index date (343.5 vs. 22.6 per 100 patient-years, P = 0.0004). Patients with more than 5 days in the hospital during the 6-month interval prior to the index date had a higher rate of transfusion than patients with 4 or fewer hospital days (478.7 vs. 38.7 per 100 patient years) (P = 0.001). With exposure adjustment, the rate of transfusion was not statistically different between groups with PVD (266 vs. 165, P = 0.40), or a history of transfusion (830 vs. 159, P = 0.17). However, patients with cardiovascular disease (CVD) at baseline had a significantly higher rate of transfusions (322.4 per 100 patient years) compared with patients without CVD (101.2 per 100 patient-years) (P = 0.04).

Multivariable logistic regression analysis estimated the likelihood of a blood transfusion adjusting for age, gender, race, history of blood transfusion, prior nephrologist visit, prior hospitalization, and comorbid conditions (Table 3). Length of follow-up was similar between transfused patients (median of 434 days, IQR of 80-735) and patients with no transfusion (466 days, IQR of 87.5-698.5, P = 0.57). Patients who were hospitalized prior to their anemia index date were 5.6 times more likely to receive a blood transfusion than patients who were not hospitalized (P < 0.0001). Age, gender, race, prior transfusion, prior nephrologist visit, diabetes, cardiovascular disease, and peripheral vascular disease were not independently significantly associated with the likelihood of transfusion after adjusting for other covariates. Cox proportional hazards regression was also conducted with the results being very similar (similar hazards as odds ratios) to the logistic regression.

**Table 3:** Likelihood of blood transfusion among non-dialysis CKD patients with persistent anemia using multivariate logistic regression

	Bivariate analysis		Multivariate logistic regression		
**Characteristics**	**Proportion with transfusion**	**P-value**	**Odds ratio**	**95% Confidence Interval**	**P-value**

**Male (ref: female)**	20% males, 20% females	0.86	0.89	0.51-1.56	0.69

**Race (ref: Caucasian)**	14%	0.054			

**African American**	25%		1.45	0.80-2.61	0.22

**Other minority race**	27%		1.80	0.48-6.68	0.38

**Age (ref: < 65 years)**	19%	0.95			

**Age 65-74 years**	20%		0.98	0.47-2.07	0.96

**Age ≥ 75 years**	21%		1.20	0.60-2.37	0.61

**Prior transfusion (ref: no prior transfusion)**	38% yes, 19% no	0.09	1.05	0.39-2.84	0.92

**Prior nephrologist visit (ref: no prior nephrologist visit)**	19% yes, 23% no	0.31	0.99	0.56-1.75	0.98

**Prior hospitalization (ref: no prior hospitalization)**	33% yes, 7% no	< 0.0001	5.64	2.90-10.96	< 0.0001

**Comorbid conditions within 6 months prior to index date**					

**Diabetes**	19% yes, 22% no	0.38	0.83	0.47-1.45	0.51

**Peripheral vascular disease**	29% yes, 18% no	0.053	1.79	0.90-3.54	0.09

**Cardiovascular disease**	24% yes, 18% no	0.14	1.02	0.56-1.83	0.96

### Transfusion characteristics

**Figure 2:**
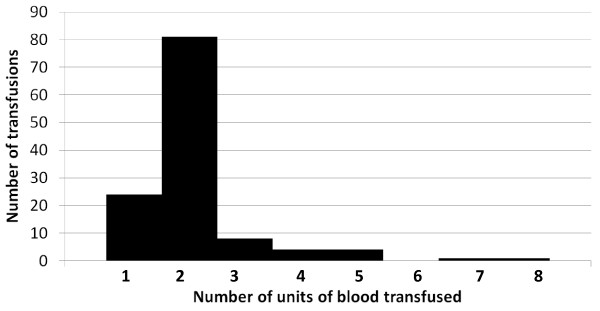
**Number of units of blood transfused per transfusion among non-dialysis CKD patients with persistent anemia**.

Approximately, 94% of transfusions occurred in the hospital inpatient setting, while 6% were administered in the ambulatory care setting. Among those patients who were transfused, the mean number of transfusions was 1.6 (SD = 1.0) and an average of 2 units of blood were transfused per transfusion. For 19.5% of the 123 transfusions, 1 unit of blood was transfused, 66% of transfusions had 2 units of blood transfused and 6.5% of transfusions had 3 units of blood transfused (Figure 2). A total of 24 patients had multiple transfusions, 18 of whom had ≥ 5 days between transfusions and 6 of whom had ≤ 4 days between transfusions.

### Trigger hemoglobin

**Figure 3:**
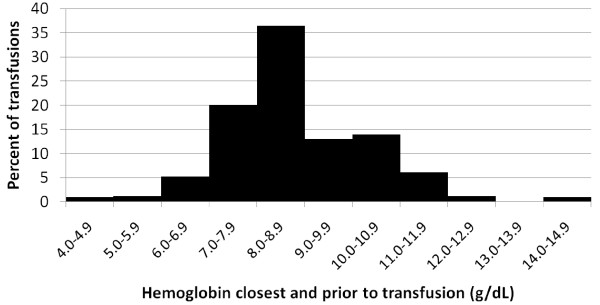
**Hemoglobin level closest and prior to the transfusion among non-dialysis CKD patients with persistent anemia**.

The hemoglobin value closest and prior to (within 2 days) each transfusion was examined and the mean Hb level was 8.8 (SD = 1.5) g/dL. Approximately 12.5% of transfusions were preceded by a Hb between 9.0 and 9.9 g/dL, 36.5% were preceded by a Hb between 8.0 and 8.9 g/dL, and 20% were preceded by a Hb between 7.0 and 7.9 g/dL (Figure 3). There were two transfusions where the closest Hb assessment was > 3 days from the transfusion and these values were not included in the analysis of trigger hemoglobin.

## Discussion

Blood transfusions were prevalent among ND-CKD patients with severe anemia recently treated within the Henry Ford Health System. Prior hospitalization consistently predicted a higher transfusion rate among these patients and patients with more days in the hospital prior to the anemia index date had more transfusions. A hemoglobin concentration around 9 g/dL was the mean level closest to the transfusion, indicative that this level may be the trigger for a blood transfusion in this population although a substantial proportion occurred at Hb > 9.0 g/dL. These findings provide new evidence of the transfusion burden among anemic ND- CKD patients treated in routine clinical practice.

A few other studies have provided estimates of transfusion frequency for anemic non-dialysis CKD patients; however, these studies included unique patient populations and less current practice trends. Lawler and colleagues [7] observed a transfusion frequency of 8.5%-15.6% among patients with Hb < 11 g/dL receiving care in the Veterans Health Administration (VHA) between 2003 and 2005. The VHA study required patients to have only 1 Hb < 11 g/dL to be included in the study. In comparison, the proportion of patients who had a transfusion of 20% in the present study may be higher due to differences in the composition of the study population; in the VHA study, 80% of patients were white, 97% of patients were men, and the definition of anemia differed compared to our study.

The present study also evaluated transfusion burden among severe anemia defined as Hb < 10 g/dL and confirmation with subsequent treatment or second Hb < 10 g/dL) non-dialysis CKD patients. A study of Medicare beneficiaries [4] between 1992 and 2004 observed an adjusted transfusion rate of 112.2 per 1000 patient-years in 2004, which is lower than the present study adjusted rate of 182.2 per 100 patient-years. The higher transfusion frequency in the present study may be due to the following factors:1) inclusion of only stage 3-5 CKD with confirmed anemia whereas the Ibrahim study [4] included all CKD stages with approximately 60% of patients without anemia, 2) inclusion of patients enrolled in a commercial health plan (HFHS managed care) whereas Ibrahim excluded commercial health plan members and focused on Medicare patients, 3) significantly larger proportion of African Americans (50%) compared with 15% in the Ibrahim study, and 4) current trends in utilization of transfusion (up to December 2009) with longer follow-up time than the 1 year point prevalence of the Ibrahim study. The HFHS did not follow a defined transfusion protocol during the time of this study but the clinical directive was to maintain Hb at 10 g/dL or higher. With the goal of maintaining Hb ≥ 10 g/dL, patients' anemia symptoms (e.g. fatigue, lack of energy) and consequences of potential allosensitization were considered in the clinical decision to transfuse CKD patients.

Based on treatment patterns observed among CKD patients treated at the HFHS, a hemoglobin level around 9 g/dL appears to trigger the majority of blood transfusions. However, approximately 21% of transfusions occurred when the closest, preceding Hb level was ≥ 10 g/dL. This may be because more than one-third (38%) of the high Hb (≥ 10 g/dL) transfusions were second and subsequent transfusions and the treating physician may not have waited for the Hb results to order subsequent transfusions given the patient's medical history. Additionally, the reasons for transfusions might not be solely based on the Hb levels; patients' symptoms will also influence physician's decisions.

A key reason for patients with CKD to avoid blood transfusions is the risk of developing allo-antibodies which prolong the time on the kidney transplant waiting list and possibly jeopardize the kidney transplant outcome [8]. In addition, blood transfusions are not without risks, including transfusion reactions, transmission of infectious agents, and iron overload [9]. Providing adequate iron stores and using ESAs, may help to reduce the need for transfusions. A better understanding of the reasons for blood transfusions may help to elucidate other pathways to reducing the transfusion burden.

Minority races (African American and other races) had a higher unadjusted rate of transfusion than Whites but after controlling for other characteristics, the adjusted transfusion rate did not differ across racial groups. This may be due to the small number of patients who received transfusions in this study; 24 Whites, 47 African Americans and 4 other races were transfused in this study.

Several study limitations should be considered. The population served by HFHS is primarily from the greater metropolitan Detroit, MI area. As such this region may not be representative of the US population and have different treatment patterns for CKD-related anemia. Thus, the study findings may be limited to non-dialysis CKD patients with anemia and not generalizable to all CKD patients. The definition for severe anemia used in the study is limited in capturing chronic, severe anemia since it used one Hb < 10 g/dL followed by treatment or a second Hb < 10 g/dL. Of the 303 patients included in the cohort with a second Hb < 10 g/dL, 60% had the second Hb level 1-2 days after the first Hb so the anemia may not be chronic or persistent for some patients. Also, only 75 patients received transfusion during the study period, which limited the likelihood to observe statistically significant differences. For example, 13 patients had a prior transfusion and only 5 of these 13 patients had a transfusion during follow-up.

## Conclusions

Blood transfusions were prevalent among ND- CKD patients with persistent anemia; 20% of patients were transfused. The mean hemoglobin level closest and prior to transfusion was around 9 g/dL and an average of 2 units of blood was transfused per transfusion. Prior hospitalization is strongly predictive of a higher transfusion rate.

## Abbreviations

CKD: chronic kidney disease; CVD: cardiovascular disease; eGFR: estimated glomerular filtration rate; ESA: erythropoiesis-stimulating agent; ESRD: end stage renal disease; Hb: hemoglobin; HFHS: Henry Ford Health System; HLA: human leukocyte antigen; ICD-9: International statistical classification of diseases and related health problems; ND: non-dialysis; PVD: peripheral vascular disease; VHA: Veterans Health Administration.

## Competing interests

This research was supported by funding from Amgen, Inc. Dr. Fox received research funds from Amgen, Inc. to conduct this research. Dr. Cong, Petersen, and Gandra are employees and stockholders of Amgen, Inc. Dr. Yee, Brooks and Lamerato have no conflicts to report.

## Authors' contributions

KMF participated in the design of the study, acquired the data, managed the data analysis and interpretation of the data, and drafted the manuscript. JY substantially contributed to the interpretation of the data and critically revised the manuscript. ZC participated in the design of the study, substantially contributed to the interpretation of the data, and helped draft the manuscript. JB assisted in the study design, performed the statistical analysis, contributed to the interpretation of the data, and critically revised the manuscript. JP participated in the study design and data interpretation, and critically revised the manuscript. LL participated in the study design, provided the data for the study, and critically revised the manuscript. SG conceived of the study, participated in the study design and critically revised the manuscript. All authors read and approved the final manuscript.

## Pre-publication history

The pre-publication history for this paper can be accessed here:

http://www.biomedcentral.com/1471-2369/13/5/prepub
